# Pure biochemicals and nanomaterials as next generation biostimulants for sustainable agriculture under abiotic stress – recent advances and future scope

**DOI:** 10.1080/15592324.2023.2290336

**Published:** 2023-12-04

**Authors:** Satish C. Bhatla, Priya Ranjan, Neha Singh, Mansi Gogna

**Affiliations:** a Department of Botany, University of Delhi, Delhi, India; b Department of Agriculture & Farmers Welfare, Ministry of Agriculture, Krishi Bhawan, New Delhi, India; c Department of Botany, Gargi College, University of Delhi, New Delhi, India; d Department of Botany, Maitreyi College, University of Delhi, Delhi, India

**Keywords:** Biostimulants, abiotic stress, nitric oxide, vitamins, melatonin

## Abstract

Sustainable agriculture faces major challenges under abiotic stress conditions owing to extensive application of chemical fertilizers which pollute water, soil and atmosphere. Biostimulants (BSs), comprising of naturally derived complex mixtures of uncharacterized biomolecules, pure biochemicals and nanomaterials, enhance nutrient use efficiency (NUE) and trigger crop’s natural defense mechanisms. While it is difficult to specify the metabolic effects of uncharacterized natural mixtures (seaweed extract, protein hydrolyzates, etc.), exogenous application of pure biochemicals and nanomaterials offers an edge as BSs since their physiological roles and mechanisms of action are decipherable. Foliar application or seed treatment of some amino acids, polyamines and biopolymers (chitosan, lipochitin oligosaccharides and thuricin 17) enable plants to overcome drought and salinity stress via activation of mechanisms for reactive oxygen species (ROS) scavenging, osmolyte regulation and chlorophyll accumulation. Interaction of nitric oxide (NO) with some vitamins and melatonin exhibits potential significance as BSs for mitigating stress by ROS scavenging and maintenance of intracellular ionic balance and membrane integrity. Near future is likely to see wide applications of nanoparticles (NPs) and nanomaterials (NMs) as BSs in view of their biphasic mode of action (bio-physical activation of membrane receptors followed by gradual release of BS into the plant cells).

## 1. Introduction

Sustainable agriculture has been facing two major challenges worldwide, namely growing population, which is expected to reach 9 billion by 2050, and climate change, leading to heat waves, rise in sea level, heavy precipitation and subsequent floods, drought and soil salinity^1,2^. All these developments directly affect crop growth and food production to a varying extent, depending on crops and their geographical locations. Excess use of chemical fertilizers to provide nutrients to the crops causes their runoff into the soil, atmosphere and water, thereby polluting the environment. Agriculture scientists have been employing the unique strategy to apply BSs in fields as stimulators of plant growth and enhancers of crop productivity, with negligible impact on the environment. According to the European Biostimulants Industry Council^3^, BSs can be defined as “*products that stimulate plant nutrition processes independent of the product’s nutrient status, with the sole aim of improving one or more of the following characteristics of the plant or its rhizosphere: (a) nutrient use efficiency (NUE), tolerance to abiotic stress, (c) quality traits and/or (d) availability of nutrients confined to soil/rhizosphere”*. Thus, BSs act by regulating plant’s natural defense mechanisms.

Current classification of BSs is based on their source of origin rather than biological activity, as follows^4^: Seaweeds and their extracts, humic and fulvic acids, protein hydrolyzates and nitrogen-containing compounds, biopolymers, inorganic compounds, beneficial fungi and bacteria. It has been observed that a combination of several BSs (vitamins, amino acids and polymers) have diverse synergistic potential and can be more effective in improving crop growth and overall productivity. However, processing of BSs as mixtures of compounds, makes isolation and characterization of active principles complex and difficult^5,6^. Use of naturally produced mixtures of uncharacterized BSs also poses problems with regard to maintenance of their compositional uniformity in the absence of standardized production under controlled conditions. Pure compounds as BSs offer several advantages over uncharacterized natural extracts and their mixtures since physiological effects of pure compounds can be easily determined. This further facilitates comprehension of their mechanism of action. BSs of pure compounds category majorly include proteinogenic and non-proteinogenic amino acids, polyamines, melatonin, pure compounds of microbial origin, biopolymers, vitamins, nitric oxide (NO) and nanomaterials. Current investigations by various research teams world over are aiming to isolate their purest active fractions, for example, the unbound fraction of humic acid has been analyzed to contain N-isopropyldecanamide (an alkamide) as the bioactive component^7^.

This review discusses the major specific roles, modes of action and possible interaction of pure biochemicals and nanomaterials as BSs, in enhancing crop productivity under abiotic stress conditions.

## 2. Potential role of amino acids and polyamines as BSs by activating ROS scavenging mechanisms and osmolyte accumulation in drought and salt-stressed crops

Among the proteinogenic amino acids, proline (Pro), methionine (met), glutamine (glu), arginine (Arg) and cysteine (Cys) have so far been found to be very effective as BSs in protecting crop plants against a variety of abiotic stress conditions. Exogenously provided amino acids as BSs (in soil or foliar applications) help plants save energy by minimizing endogenous synthesis of amino acids, thereby enhancing their stress tolerance ability. Pro is one of the most efficient essential amino acids used as a BS in various crops facing drought stress, which subsequently exhibit improved water status and enhanced activities of various ROS scavenging enzymes^8^. Foliar application of Pro also improves nutrient uptake from soil. Similarly, Met is another component of many stress-related proteins and it increases tolerance to drought and salt stress in cowpea and tomato, respectively, by regulating plant redox state and osmolyte content, thereby limiting damage to various biomolecules^9,10^. Methionine sulfoxide reductase (MSR) controls the redox state of Met, and is crucial in Met-mediated defense mechanisms against stress^11^. Glu enhances antioxidant protection against abiotic stress in crop plants, such as soybean, by increasing the levels of superoxide dismutase (SOD) and catalase (CAT)^12^, and subsequently, a rise in the levels of compatible osmolytes is noted^13^. Likewise, Cys, a precursor of the tripeptide antioxidant glutathione (GSH), also provides protection against oxidative stress in soybean. Cys, alongside Arg, plays a crucial role in amelioration of stress due to heavy metals in plant tissues^12,14^. Plants also produce a number of non-protein amino acids (NPAA) whose exogenous application as a BSs generates salt tolerance via maintenance of membrane integrity, stabilization of enzymes, osmoregulation and defense by antioxidant systems. Glycine betaine (GB), gamma-aminobutyric acid (GABA) and beta-aminobutyric acid (BABA) are three major NPAA ubiquitous in plants whose foliar or root application protects crop plants against drought and salt stress by enhancing the transcription of genes associated with photosynthetic activity and antioxidant defense^15^.

A number of biogenic amines, primarily putrescine (Put), spermidine (Spd) and spermine (Spm), have been observed to serve as BSs owing to their ability to function as osmoprotectants under a wide range of biotic and abiotically stressful conditions, by regulating seed germination and subsequent growth and developmental processes. Polyamines (PAs) modulate developmental outlines by regulating replication, transcription, translation, cell division and stabilization of cell membrane and cell wall. Foliar administration of PAs enhances tolerance to drought and salinity in wheat, lettuce and guava by prevention of chlorophyll degradation and uptake of noxious uptake of Na^+^ and Cl^−^ by plants^16–18^. In tomato plants, Put reduces electrolyte leakage under cold stress^19^ Foliar application of Spm and Spd protects wheat and soybean plants from drought stress and tomato seedlings from salt stress by reducing accumulation of ROS, improvement of Na^+^/K^+^ ratio and increasing osmolyte accumulation^20–22^. Catabolism of PAs produces H_2_O_2_, which acts as a stress signaling molecule and induces ROS-dependent protective mechanisms, as osmoprotectants, transcriptional regulators, ion channels regulators and in PCD^23^.

**Figure 1.**
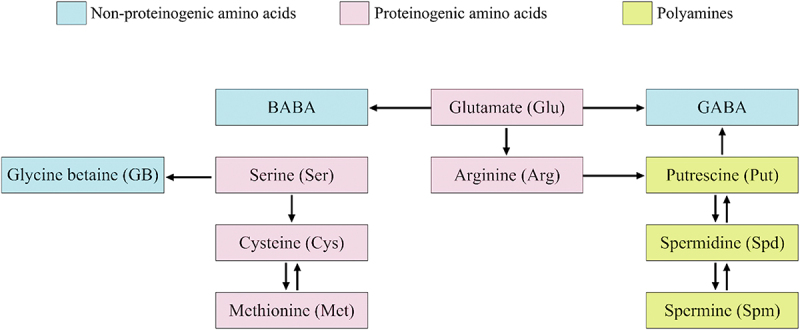
Interactive action of proteinogenic/non-proteinogenic amino acids and polyamines as BS_s_ in crops. Abbreviations – β-aminobutyric acid (BABA); γ-aminobutyric acid (GABA).

A clear metabolic interrelationship among some of the key proteinogenic amino acids, non-proteinogenic amino acids and polyamines makes it evident that any of the compounds from these categories is likely to impact plant growth and possibly improve their survival under abiotic stress conditions, either independently or through modulation of one or more of the metabolically regulated biomolecules (Figure 1). These observations, therefore, provide interesting directions for future research leading to better understanding of their roles as BSs.

## 3. Biopolymers as upcoming BSs

In the recent past, some polysaccharides and polypeptides/peptides have found uses as BSs, largely against drought stress. These include chitosan, alginate oligosaccharides (AOS), lipochitooligosaccharides (LCO), poly(y-glutamic acid) [y-PGA] and thuricin 17 peptide. Chitosan, a linear polysaccharide, is deacylated chitin (poly B-1,4-D-glucosamine). It is used as a biocompatible, antibacterial environment friendly polyelectrolyte, capable of forming gels with multivalent anions. Commercially, it is obtained from the acid hydrolysis of crustaceans (such as shrimps and crabs) or from the cell walls of mushrooms. Alginate oligosaccharides (AOS) are obtained from marine brown algae. Y-PGA, a polypeptide composed of D- and L-glutamic acid monomers, is obtained from microbial fermentation. LCO is commercially obtained from *Bradyrhizobium japonicum* while Thuricin 17 is derived from *Bacillus thuringiensis*. Foliar spray and seed treatment of these biopolymers confirm the efficacy of these BSs as mitigators of drought stress in maize, barley, wheat, cucumber and Brassica^24–27^. Biopolymers broadly function by improving chlorophyll content, activating ROS scavenging system and osmolyte accumulation. In addition to their roles as BSs by direct application, alginates and chitosan are also used to encapsulate pesticides and fertilizers.

## 4. Nitric oxide- its independent roles as BS

Nitric oxide (NO) being an established redox signaling biomolecule, is expected to overcome the impact of abiotic stress conditions, which often results in a stark rise in endogenous ROS levels in plants. Salt stress affects plant development by regulating photosynthesis, antioxidative defense mechanisms and ionic homeostasis. NO reverses the adverse effects of salinity stress by stimulating K^+^ accumulation in the cytosol of wheat, proline accumulation in tomato, enhancing the activity of antioxidative enzymes in rapeseed and enhancing the activity of H^+^-ATPase^28^. Additionally, NO can regulate the accumulation of compatible osmolytes, such as glycine betaine (GB), in plants under condition of salt stress by regulating the activity of their key biosynthetic enzyme, betaine aldehyde dehydrogenase (BADH)^29^. The likelihood of complex formation between GB – NO is high, and could be responsible for the resultant elicitation of physiological responses via modulation of BADH transcript levels. These findings hold significant promise for upscaling the use of NO donors as potential foliar sprays in agricultural fields. Choice of NO donor is crucial since sodium nitroprusside (SNP) is no more considered the right option due to simultaneous release of cyanide and iron (along with NO) in its aqueous solutions, thereby causing interference in its physiological effects^30^. Diethylenetriamine NONOate (DETA) has been found to release 16% more NO than SNP in aqueous solution and is more effective in sunflower seedling^31^.

## 5. Vitamins and their crosstalk with NO

Application of both water-soluble and fat-soluble vitamins mediates enhanced tolerance to drought stress in plants^2^. Water soluble vitamins, namely B1 (thiamine), B6 (pyridoxine), B9 (folic acid), C (ascorbic acid), and fat-soluble vitamins, namely pro-vitamin A (carotenes), E (tocopherol) and K1 (phylloquinone) exhibit tremendous ability to quench various ROS produced as stress response. Vitamin B3 also increases accumulation of photosynthetic pigments and proline, and reduces Na^+^/K^+^ ratio. Vitamin E reduces lipid peroxidation and protects membrane integrity in addition to accumulation of proline and photosynthetic pigments. Possible interaction of some of the vitamins with NO offers novel mechanisms of their action as ROS scavengers in plants facing stress^32^. Out of the two oxidation states of cobalamin (cbl) in vitamin B12 (cyanocobalamin) [cbl(II) and (III)], cbl (III) reacts with NO in a two-step process and reduces cbl (IIII) to cbl (II). The cbl (III)-NO complex can also transfer its NO moiety to hemoglobin (Hb) and glutathione. Likewise, reduced L-ascorbic acid (vitamin C) interacts with nitrosating species (such as NO^+^, N_2_O_3_, and S-nitrosothiol), though it does not react with NO directly. The oxidized form of ascorbate (dehydroascorbate) undergoes spontaneous decay to ascorbyl radical, which can combine with NO to form O-nitrosoascorbate. The latter undergoes hydrolysis to ascorbate and NO_2_. Thus, ascorbate anion is a valuable marker of oxidative stress

## 6. Melatonin as a BS functions as an antioxidant and as a NO carrier

Melatonin, a tryptophan derivative, is an established growth regulator in plants^33^. As a BS, effect of melatonin has so far mostly been examined in laboratory conditions, which holds a great potential for use in field. Exogenous application of melatonin at specified concentrations alleviates drought stress in maize and kiwi seedlings^34,35^, salinity stress in cucumber and strawberry^36,37^ and chilling stress in tomato^38^. The protective role of melatonin is primarily attributed to its strong ability to quench ROS as well as stimulate the activities of ROS scavenging enzymes (CAT, SOD, GPX, POD). Additionally, it is also known to enhance photosynthesis by modulating carotenoid accumulation, as also increase stomatal conductance in some of the above-stated crop plants. Recently, work undertaken in the corresponding author’s laboratory also demonstrated molecular interaction of melatonin with nitric oxide, resulting in the production nitrosomelatonin (NOMela)^39–42^. NOMela has been observed to get transported over transcellular distances within the plant. These observations highlight the possible physiological significance of NOMela in ROS scavenging mechanisms operative in plants, particularly under abiotic stress conditions.

## 7. Alkamides – the active ingredient of humic acid now in use as a pure BS

Recent NMR spectroscopic and GC-MS analysis of uncharacterized but extensively used BS, humic acid (HA), found prominently in cattle manure and vermicompost, has lead to the discovery of a new class of growth regulator (an alkamide) as its major bioactive ingredient^7^. Both, the bioactive alkamide purified from HA (N-isopropyldecanamide) and also synthesized, enhance root proliferation, H^+^-ATPAse activity and NO accumulation in maize seedling roots. It is expected that as a result of the stimulation of plasma membrane associated H^+^-ATPase activity, seedling roots enable activation of ion channels to facilitate greater mobilization of mineral ions from soil. Enhanced NO accumulation in the seedling roots is expected to be involved in auxin-mediated root proliferation. Thus, alkamides are the major bioactive constituents of HA and seem to operate through auxin-independent and auxin-dependent signaling roots to promote mineral ion uptake from the rhizosphere and root growth. These significant findings are expected to encourage scientists to examine the impact of alkamides on various crops in laboratory and field conditions.

## 8. Antitranspirants serve as BS through their physical and physiological actions

A variety of nontoxic compounds used as foliar spray on crops, are known to reduce transpiration, thereby reducing water loss and irrigation requirements, thus enhancing crop productivity under drought conditions. Antitranspirants (Ats) can be considered under four broad categories namely, metabolic, reflective, film-forming and growth retardants^43^. The metabolic Ats include ABA (or pyrabactine-asynthetic agonist of ABA), chitosan [poly(D-glucosamine)], fulvic acid and phenyl mercuric acid (PMA). The pivotal role of metabolic antitranspirants is via closing of the stomatal pore, induction of enzymatic antioxidant system and maintenance of membrane integrity. The reflective Ats include aluminosilicate (Kaolin), calcium carbonate, calcium oxide, magnesium carbonate and magnesium silicate (Florisil). They function by increasing light reflection by the leaves, drop in leaf temperature and reduction in overall transpiration rate. Common examples of film-forming Ats are di-1-p-menthane (obtained from pine resin), poly-1-p-menthane, acrylic polymers, silicone oil (e.g. polydimethylsiloxane) and plastic films (polyethylene resin). They majorly function by blocking stomatal pores under conditions of stress or exogenous application. Among the growth retardant category of Ats, cycocel (chlormequat chloride) is known to increase water use efficiency of crops, reduce shoot growth and increase root growth. Among all these Ats, ABA, kaoline and di-1-p-menthene are considered as low-risk BSs. Some others, such as PMA, can, however, be toxic to fruits and vegetables. Many Ats work as BSs only at low temperature and block gaseous exchange from leaves (waxes and other film-forming Ats). They are, therefore, not suitable as BSs except when crop survival is at stake since they also reduce photosynthesis. They can also reduce mineral uptake by plants and can be used only for brief period of crop growth during the phase of maximum moisture sensitivity of plants. The film-forming Ats are recommended for not prolonged use and their effect as BSs is long lasting, hence detrimental.

## 9. Nanoparticles and nanomaterials as future BSs

**Figure 2.**
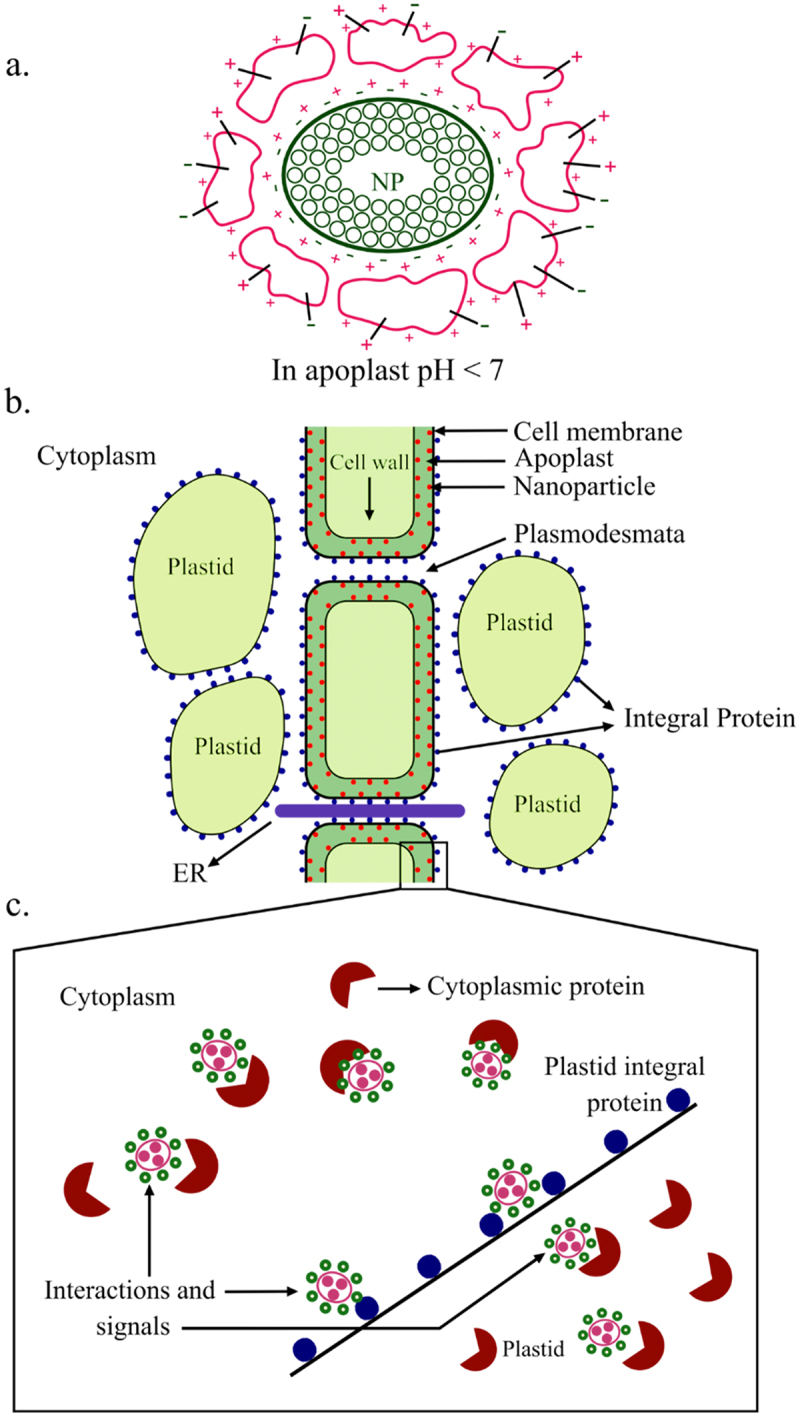
(a) the electrostatic interaction between positive charges of cell surface proteins and negative charges of NPs (nanoparticles), resulting in ‘corona’ formation at the apoplastic pH of less than 7. (b) NPs bind the cell surface via charged groups on the cell surface of ‘corona’ and enter the cell by endocytosis or through pores. (c) Once internalized, NPs interact with cytoplasmic proteins and organelles, bringing about metabolic adjustments and changes in gene expression, thereby improving growth.

Since nanoparticles (NPs) and nanomaterials (NMs) can enhance plant growth in low concentations, they may be considered as BSs. The biphasic biostimulatory action of NPs and NMs sequentially involves their physico-chemical interactions with plant cell surface and cell membrane, leading to activation of receptors and transporters for influx of mineral ions. This is followed by the release of chemical elements from the NPs or NMs, which trigger a variety of biochemical processes supporting growth^44^. Physio-chemical interaction of NPs and NMs (first phase of their action) occurs irrespective of their own composition but it is determined by pH, ionic strength and other dynamic properties of the cell wall and cell membrane. It is the net negative charge on the cell surface which determines the impact of the interaction of NPs and NMs with ions present in the apoplast. The charge on cell surface is determined by the activity of PM-associated integral and peripheral proteins and flow of protons from proton pumps. Likewise, the metabolism-related and response-related proteins determine the charge status on the cell wall. They offer molecular interplay sites for NPs and NMs. When in contact with plant cell surface, NPs and NMs adsorb biomolecules such as peptides and proteins, forming a corona with lowest free energy (Figure 2). This serves as a mechanism for decreasing NP’s or NM’s surface free energy regardless of their character (cationic, anionic or neutral). The corona composition gets modified as NPs migrate from environment to another. NPs and NMs migrate toward cell surface by diffusion, and they not probably have any specific receptors.

The biostimulatory action of NPs and NMs is due to their high surface/volume value and surface charge density. They modify the transmembrane electric potential by their interaction with charges on the surface of cell wall and cell membrane, thereby altering modulating the transport of ions and metabolites across the cell wall and PM. Additionally, NMs and NPs could bring about conformational changes in the enzymes coming in their contact. After the primary response (changes in surface charges, followed by internalization of NPs in the cells by endocytosis or pore formation), subsequent biochemical actions (secondary response) modify the cellular metabolism, causing biostimulation of plant growth. NPs of titanium dioxide (TiO_2_) have been used to promote antioxidant activity and enhanced stress tolerance to cold, drought, NaCl and UV radiation in plants such as chickpea, tomato, flax and basil^44^. Since TiO_2_ is poorly soluble in water, its response as a BS is probably due to surface interaction only. Cu nanoparticles are beneficial for plant growth at concentrations much lower than Ti. Foliar spray of NPs and NMs is preferred for best results as they interact with leaf cuticle in tomato, cucumber and mung bean. The definition of BSs does not include those fertilizers which contribute essential elements. Ti NPs and Cu NPs do not directly contribute the respective elements since their suspension releases only up to 0.5% of the ions in case of Cu NP. Nanourea cannot be considered as a BS since it releases almost up to 89% of its weakly bound urea from the urea-coated amorphous calcium phosphate nanoparticles (ACP NPs)^45^.

## 10. Conclusion and perspectives

The major advantages of using pure compounds as BSs are due to the possibility of quality control and standardization of their formulations. Thus, their protective actions and mechanisms of operation can be clearly defined. It is also possible to metabolically assess possible biochemical interactions among some of them, such as among amino acids and polyamines, as also a crosstalk of NO with some of the vitamin (B12 and C) or with melatonin. Thus, accurate information on the effects of pure compounds as BSs is achievable through metabolomics, proteomics and transcriptomics. The use of pure biochemicals as BSs is, however, still at an early stage and holds tremendous potential for field applications on various crops under stressful growth conditions, leading to better crop yield in a precisely controlled manner.
